# Transcriptomics Study to Determine the Molecular Mechanism by which sIL-13R*α*2-Fc Inhibits Caudal Intervertebral Disc Degeneration in Rats

**DOI:** 10.1155/2020/7645989

**Published:** 2020-08-14

**Authors:** Xin Wang, Jianshi Tan, Junhao Sun, Pengzhong Fang, Jinlei Chen, Wen Yuan, Huajiang Chen, Yang Liu

**Affiliations:** ^1^Department of Orthopedics, First Clinical Medical College of Lanzhou University, The First Hospital of Lanzhou University, Lanzhou, Gansu, China 730000; ^2^Changzheng Orthopedics Hospital, Second Military Medical University, Shanghai, China 200003

## Abstract

**Background:**

Intervertebral disc degeneration is related to tissue fibrosis. ADAMTS can degrade the important components of the ECM during the process of intervertebral disc degeneration, ultimately resulting in the loss of intervertebral disc function. sIL-13R*α*2-Fc can inhibit fibrosis and slow down the degeneration process, but the mechanism involved remains unclear.

**Objective:**

To determine the mechanism by which sIL-13R*α*2-Fc inhibits ECM degradation and reduces intervertebral disc tissue fibrosis using a transcriptomics analysis.

**Methods:**

A rat model of caudal intervertebral disc degeneration was established, and Sirius red staining was used to observe the pathological changes in the caudal intervertebral disc. Transcriptome sequencing was employed to assess the gene expression profiles of the intervertebral disc tissues in the model group and the sIL-13R*α*2-Fc-treated group. Differentially expressed genes were identified and analyzed using GO annotation and KEGG pathway analyses. Real-time fluorescence quantitative PCR was used to verify the expression levels of candidate genes. The levels of GAG and HA were quantitatively assessed by ELISA, and the levels of collagen I and collagen II were analyzed by western blotting.

**Results:**

Sirius red staining showed that in the model group, the annulus fibrosus was disordered, the number of breaks increased, and the type I collagen protein levels increased, whereas in the sIL-13R*α*2-Fc group, the annulus fibrosus was ordered, the number of breaks decreased, and the type II collagen protein levels increased. In comparison with the model group, we identified 58 differentially expressed genes in the sIL-13R*α*2-Fc group, and these were involved in 35 signaling pathways. Compared with those in the model group, the mRNA expression levels of *Rnux1*, *Sod2*, and *Tnfaip6* in the IL-13R*α*2-Fc group were upregulated, and the mRNA expression levels of *Aldh3a1*, *Galnt3*, *Fgf1*, *Celsr1*, and *Adamts8* were downregulated; these results were verified by real-time fluorescence quantitative PCR. TIMP-1 (an ADAMTS inhibitor) and TIMP-1 combined with the sIL-13R*α*2-Fc intervention increased the levels of GAG and HA, inhibited the expression of type I collagen, and promoted the expression of type II collagen.

**Conclusion:**

*Adamts8* may participate in the degradation of ECM components such as GAG and HA and lead to an imbalance in the ECM of the intervertebral disc, resulting in intervertebral disc degeneration. sIL-13R*α*2-Fc promoted anabolism of the ECM and increased the levels of ECM components by inhibiting the expression of *Adamts8*, thus maintaining the dynamic equilibrium of the ECM and ultimately delaying intervertebral disc degeneration.

## 1. Introduction

Intervertebral disc degeneration is the underlying basic pathological process of a series of spinal degenerative diseases (such as disc herniation, spinal canal stenosis, spondylolisthesis, spinal instability, and neuropathy) [1]; however, the specific pathophysiological mechanism involved remains unclear. Most scholars believe that the main mechanisms of intervertebral disc degeneration include tissue fibrosis, intervertebral disc cartilage endplate degeneration, a decrease in the number of bone marrow cells (either through increased apoptosis and autophagy or by decreased proliferation), local effects (such as oxidative stress and vascular hyperplasia), decomposition of the extracellular matrix, and the effects of inflammatory cytokines [2–4]. Because of the changes in the rates of the synthesis and decomposition of the extracellular matrix in degenerative discs, there are increased levels of type I collagen and decreased levels of type II collagen, and proteoglycan synthesis is reduced. The degree of nucleus pulposus cell apoptosis, which is considered to be the general pathophysiological process that occurs in intervertebral disc degeneration, is also increased [5]. The extracellular matrix provides mechanical support and physical strength for the integrity of tissues and organs as well as the entire body, thereby playing a prominent role in wound repair and fibrosis [6, 7]. An imbalance between ECM synthesis and degradation can lead to excessive ECM deposition, resulting in fibrosis [8]. Fibrosis is a common underlying pathological change that occurs in many chronic noninfectious diseases, and it is the main cause of disability and death resulting from many chronic diseases and involves almost all organs and systems in the human body [9].

The occurrence and development of fibrosis are closely related to many factors, particularly cytokines [10]. IL-13 and its receptor IL-13R*α*2 have become “hot” molecules in asthma research as well as in studies of other fibrotic diseases in recent years[11]. IL-13, a crucial regulator of the fibrotic extracellular matrix, is produced by activated Th2 cells and has been proven to play a role in many inflammatory and fibrotic diseases, such as idiopathic pulmonary fibrosis and systemic sclerosis [12, 13]. IL-13 increases TGF-*β* levels by acting through its receptor IL-13R*α*2, thereby inducing fibrosis [14]. It has been found that overexpression of the high-affinity antagonist IL-13R*α*2, which is related to IL-13R*α*1 and can bind IL-13 but lacks any signaling capability, inhibits the fibrotic markers induced by IL-13 *in vitro* and in bleomycin-induced pulmonary fibrosis, indicating that IL-13R*α*2 has antifibrotic properties [15]. A recombinant soluble interleukin-13 receptor *α*2 fusion protein (sIL-13R*α*2-Fc) has been generated and shown to also have a high affinity for IL-13, and accordingly, it acts as an IL-13 antagonist to block the profibrotic effects of IL-13 and reduce the deposition of abnormal ECM in injured tissues [16, 17]. Thus, some scholars have proposed that IL-13R*α*2 may delay the progression of intervertebral disc degeneration, which has been confirmed by our previous research. We found that intervertebral disc degeneration involves tissue fibrosis. The recombinant sIL-13R*α*2-Fc fusion protein was found to regulate the expression levels of types I and II collagen by increasing the levels of GAG, CS, KS, and HA, thereby slowing down the process of intervertebral disc degeneration [18]. Nevertheless, the mechanism by which sIL-13R*α*2-Fc delays intervertebral disc degeneration remains unclear. To clarify this issue, we used transcriptomics technology to investigate the molecular mechanism by which sIL-13R*α*2-Fc delays intervertebral disc degeneration at the gene transcriptome level.

## 2. Materials and Methods

### 2.1. Establishment of a Rat Model with Caudal Intervertebral Disc Degeneration

In this study, all the animal experiments were approved by the Animal Experiment Ethics Committee of the First Hospital of Lanzhou University, Gansu, China. The rats used in the study were SD rats (36 rats, aged 8–10 weeks, weighing 250–300 g) purchased from Lanzhou Veterinary Research Institute of Chinese Academy of Sciences (Lanzhou, Gansu, China). The rat model with caudal intervertebral disc degeneration was established following the experimental method of Chia-Hsian et al. [19] The rats were anesthetized with sodium pentobarbital (1%, 6 mL/kg), the tails of the rats were cleaned, and the limbs of the rats were fixed in the prone position. A 20 G puncture needle was used to puncture the intervertebral space between C7/8 and C8/9 vertically; it was gently rotated 360° and pulled out after 30 seconds, with the puncture depth being 3 mm. One week after successful puncture, the SD rats were randomly divided into five groups: a blank control group, a sham group, a model group and a 2 mg/kg sIL-13R*α*2-Fc group, a 1 mg/kg TIMP-1 (ADAMTS inhibitor) group, and a TIMP-1+2 mg/kg sIL-13R*α*2-Fc group, with six rats in each group. The model group was given an equivalent volume of normal saline.

### 2.2. Sirius Red Staining

After 8 weeks of sIL-13R*α*2-Fc intervention, three rats were randomly selected from every group for sacrifice. The caudal intervertebral disc tissues of the rats were stripped and fixed with 4% paraformaldehyde, and 6 *μ*m-thick paraffin-embedded sections were generated. After staining with picric acid-Sirius red (Zhongshan Reagent Co., Ltd.) for 30 min, the sections were dehydrated using a stepwise gradient of increasing ethanol concentrations, made transparent with xylene, and then sealed with neutral gum. A polarized light microscope (Olympus BX51) was used to observe the distributions of type I and type II collagen.

### 2.3. RNA Extraction and Transcriptome Sequencing

After 8 weeks of sIL-13R*α*2-Fc intervention, the intervertebral disc tissues of three rats were taken from both the model group and the sIL-13R*α*2-Fc group, and total RNA was extracted using RANiso Plus (Takara, Japan) reagent. The concentration of RNA was measured with a NanoDrop 2000 device (Thermo), and the integrity of the RNA was assessed using an RNA Nano 6000 analysis kit (Agilent Technologies, CA, USA). Qualified RNA samples were submitted to Shanghai Baipu Biotechnology Co., Ltd. for transcriptome sequencing.

### 2.4. Differential Expression Analysis

Fragments per kilobase of transcript per million (FPKM) was used to standardize the gene expression levels, and the differential fold change (FC) in expression was calculated based on the FPKM value. DEseq was employed to analyze the difference in gene expression among different samples and to adjust the *P* value to strictly control the false discovery rate (FDR). Differentially expressed genes were screened using the adjusted *P* < 0.01 and log_2_FC ≥ 2.

### 2.5. Enrichment Analysis of Differentially Expressed Genes

The DAVID 6.8 online analysis tool (https://david.ncifcrf.gov/home.jsp) was adopted to analyze the enrichment of differentially expressed genes, including Gene Ontology (GO) and Kyoto Encyclopedia of Genes and Genomes (KEGG). With *P* < 0.05 indicating significant enrichment, the number of differential genes in each GO term or pathway was counted. The functions and main pathways of differentially expressed genes were determined by GO and KEGG significant enrichment analyses.

### 2.6. Verification of Candidate Genes

Based on the results of the transcriptome bioinformatics analysis, differentially expressed genes that may be involved in the mechanism by which sIL-13R*α*2-Fc protects the intervertebral disc from degeneration were identified. Two of these, namely *Fgf1* and *Adamts8*, were confirmed by real-time quantitative PCR with *GAPDH* as the housekeeping gene. The primers used are listed in Table 1. The cDNA samples from the intervertebral disc tissues were used as templates, and the PCR verification was carried out using the Bio-Rad CFX 1000 Real-Time PCR system. Each sample was assessed in triplicate.

### 2.7. ELISA Analysis

According to the aforementioned modeling method, the rat model of intervertebral disc degeneration was reestablished, and the rats were regrouped: a blank control group, a sham group, a model group, a 2 mg/kg sIL-13R*α*2-Fc group, a 1 mg/kg TIMP-1 (ADAMTS inhibitor) group, and a TIMP-1+2 mg/kg sIL-13R*α*2-Fc group, with six rats in each group. The blank group and model group were given an equivalent volume of normal saline. After 8 weeks of drug intervention, the intervertebral disc tissues of the rats were homogenized in PBS buffer, and the homogenate was centrifuged at 5000 × *g* for 5 min to generate a supernatant for analysis. Following the instructions for the rat GAG ELISA Kit (mlbio, ml059570) and the HA ELISA Kit (mlbio, ml059167), each antibody was diluted with 0.05 M pH 9 osmium carbonate coating buffer, and blank, standard, and sample wells were prepared, after which samples were added to the appropriate wells. Then, 0.1 mL of enzyme-labeled antibody was added to each reaction well followed by incubation at 37°C for 0.5 h. After the substrate reaction solution (TMB) was added to each well and allowed to incubate for 10–30 min, the terminating reaction solution was added to terminate the reaction. The OD value was measured at 490 nm with a microplate reader (iMark 19718, Bio-Rad, Hercules, CA, USA).

### 2.8. Western Blot

After 8 weeks of drug intervention, 20 mg of intervertebral disc tissue was taken from the blank control group, the model group, the TIMP-1 group, and the TIMP-1+2 sIL-13R*α*2-Fc group and lysed with RIPA (Applygen, YZ-C1053) buffer. The total protein extracted from the rat intervertebral disc tissues was then quantified by the bicinchoninic acid (BCA) method. The tissue lysate samples were loaded onto an SDS-PAGE gel and separated by electrophoresis. Following electrophoretic transfer to a polyvinylidene fluoride (PVDF) membrane, the membrane was blocked with 5% skimmed milk for 1 h and then incubated overnight at 4°C with specific primary antibodies, including an anti-collagen I antibody (ab34710, 1 : 1000, Abcam), an anti-collagen II antibody (ab34712, 1 : 1000, Abcam), and an anti-*β*-actin antibody (ab8227, 1 : 2000, Abcam). The membrane was then incubated with a horseradish peroxidase- (HRP-) conjugated secondary antibody (E030110, 1 : 5000, Earthhox) for 1.5 h at room temperature. Protein signals were detected using ECL chemiluminescent agents and then quantified by densitometry using Image-Pro plus 6.0 software.

### 2.9. Statistical Analysis

SPSS19.0 was employed for statistical analysis of the experimental data following the univariate analysis method, and the results of pairwise comparisons between groups are presented as $\bar{x}\pm \text{SE}$; *P* < 0.05 was used to indicate that a difference was statistically significant.

## 3. Results

### 3.1. Sirius Red Staining of the Caudal Intervertebral Disc in Rats

**Figure 1:**
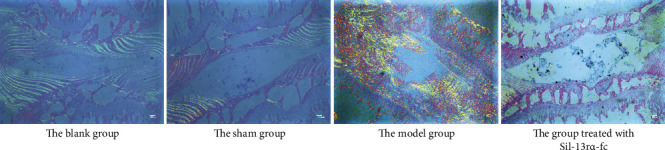
Sirius red staining showing the effect of sIL-13R*α*2-Fc on the intervertebral disc tissue in rats.

We performed a Sirius red staining analysis of the intervertebral disc tissue of rats after the 8th week of the sIL-13R*α*2-Fc intervention, and the results are shown in Figure 1. The intervertebral disc tissues in the blank group and sham group mainly consisted of type II collagen, followed by type I collagen, with a neatly arranged annulus fibrosus. Compared with that in the sham group, the intervertebral disc tissue in the model group was mainly composed of type I collagen, with a disordered annulus fibrosus and an increased number of breaks and obvious signs of degeneration. Treatment with sIL-13R*α*2-Fc significantly decreased the extent of intervertebral disc degeneration in rats, which was characterized by the dominant expression of type II collagen, an evenly arranged annulus fibrosus, and a decreased number of breaks. These data indicate that sIL-13R*α*2-Fc can slow down the process of disc degeneration.

### 3.2. Differential Expression Analysis

**Figure 2:**
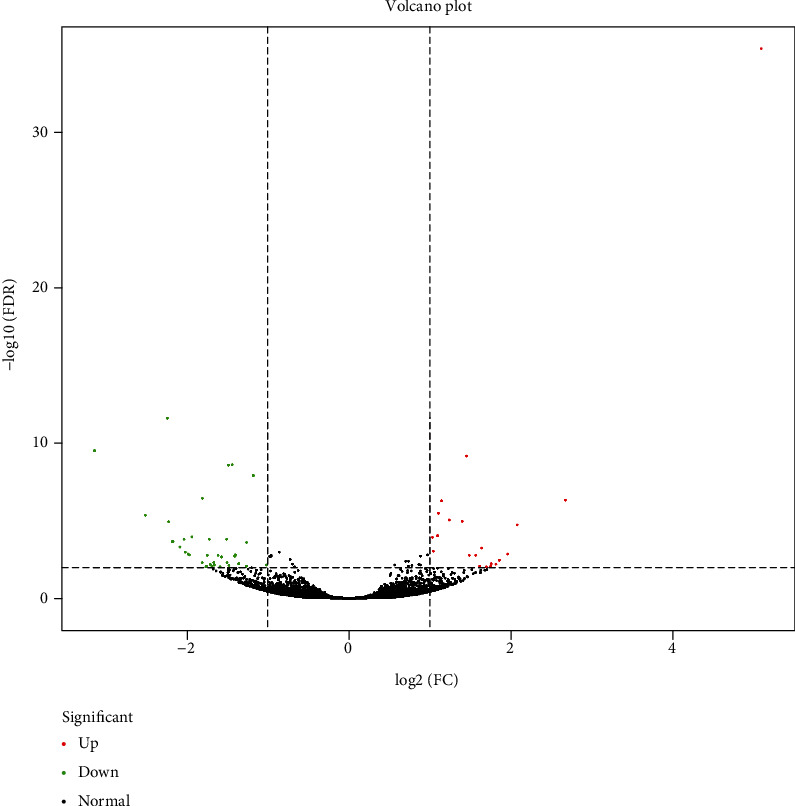
Volcano plot of differentially expressed genes. Notes: each point in the differential expression volcano plot represents a gene. The abscissa represents the absolute value of the fold change in gene expression in the two samples, and the ordinate represents the statistically significant negative logarithm value of the gene expression change. In the plot, green dots represent downregulated differentially expressed genes, red dots represent upregulated differentially expressed genes, and black dots represent nondifferentially expressed genes.

The FPKM method was used to determine the gene expression patterns in the model group and the sIL-13R*α*2-Fc group, after which DEseq was used to identify differentially expressed genes in the caudal intervertebral disc of SD rats. A total of 58 differentially expressed genes were identified between the model group and the sIL-13R*α*2-Fc group, of which 22 genes were upregulated and 36 genes were downregulated (Table 2). The volcano plot in Figure 2 presents the distribution of the upregulated and downregulated genes.

### 3.3. GO Classification of Differentially Expressed Genes

**Figure 3:**
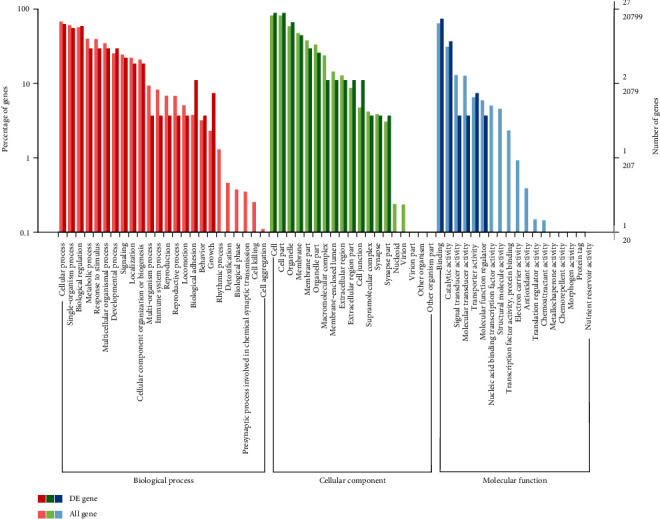
Statistical analysis of the GO annotation classification of differentially expressed genes. Notes: the abscissa represents the GO classification; the left side of the ordinate is the percentage of the number of genes, and the right side is the number of genes.

The differentially expressed genes were functionally annotated according to the GO classification criteria. This analysis revealed that 42 of the differentially expressed genes in the sIL-13R*α*2-Fc group could be classified into the three GO branches, namely biological process, molecular function, and cellular components. The 42 differentially expressed genes were further annotated into 61 different subsets of functions under the three major GO branches, including 24 biological process functions, 18 molecular functions, and 19 cellular component functions (Figure 3). The differentially expressed genes were primarily associated with the processes of cell regulation, metabolism, behavior, biological regulation, and biological genes.

### 3.4. KEGG Pathway Enrichment Analysis of Differentially Expressed Genes

**Figure 4:**
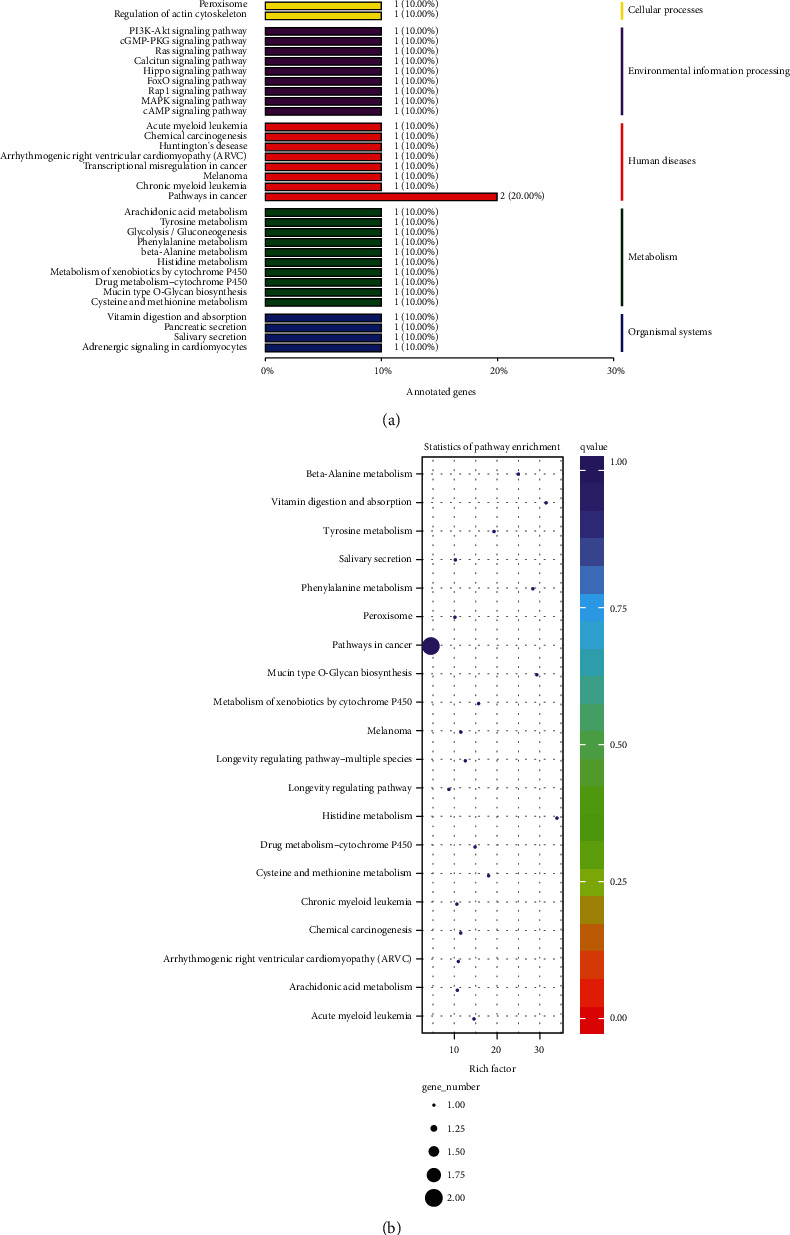
Scatter plot of the KEGG pathway enrichment for differentially expressed genes. Notes: each circle in the figure represents a KEGG pathway; the ordinate is the name of the pathway, and the abscissa is the enrichment factor, which represents the ratio of the proportion of genes annotated to a pathway for differentially expressed genes to the proportion of genes annotated to that pathway for all genes.

In the KEGG pathway analysis, compared with the model group, 26 genes in the sIL-13R*α*2-Fc group were upregulated or downregulated, involving 35 metabolic pathways (Figure 4(a)). Among them, the PI3K/Akt, Ras/MEK/ERK, and MAPK signaling pathways were involved in the prevention and treatment of intervertebral disc degeneration by sIL-13R*α*2-Fc; among these three, pathways involved in cancer (2 genes) contained the most abundant genes. As shown in the enrichment figure from the KEGG pathway analysis, the differentially expressed genes in the intervertebral disc tissue of SD rats in the sIL-13R*α*2-Fc group were mainly enriched in the following pathways: histidine metabolism, vitamin digestion and absorption, mucin type O-glycan biosynthesis, and phenylalanine metabolism (Figure 4(b)), representing the top 20 pathways with the least significant *Q* values.  Table 3 in the pathways with a higher differential gene enrichment rate in the disc tissue of the sIL-13R*α*2-Fc group than in the model group. After the intervention with sIL-13R*α*2-Fc, the expression levels of *Runx1*, and *Sod2*, were upregulated, whereas those of *Aldh3a1*, *Galnt3*, *Fgf1*, Atp2b4, Mri1, Dsc2, Mmachc, Ptgds and *Adamts8* were downregulated.

### 3.5. Real-Time Fluorescence Quantitative PCR Verification

**Figure 5:**
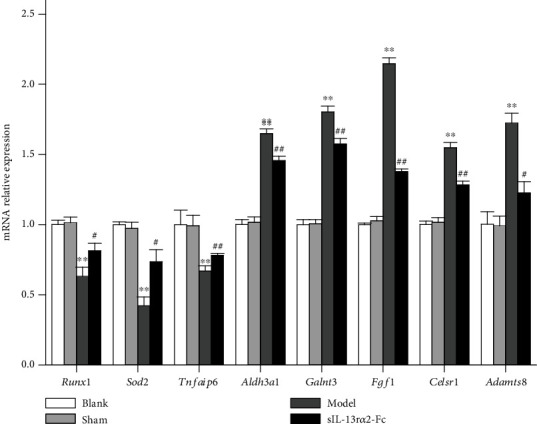
Real-time fluorescence quantitative PCR verification of genes. vs. the sham group, ^∗∗^*P* < 0.01; vs. the model group, ^#^*P* < 0.05, ^##^*P* < 0.01.

To verify the transcriptional levels of the genes identified in the transcriptome analysis to be important in the mechanism by which sIL-13R*α*2-Fc protects against rat intervertebral disc degeneration and to confirm the accuracy of the bioinformatics analysis, we used real-time fluorescence quantitative PCR to verify the expression of the candidate genes *Runx1*, *Sod2*, *Tnfaip6, Aldh3a1*, *Galnt3*, *Fgf1*, *Celsr1*, and *Adamts8*. The results are shown in Figure 5. Compared with those in the sham group, the expression levels of *Runx1*, *Sod2*, and *Tnfaip6* in the model group decreased significantly (*P* < 0.05), whereas the expression levels of *Aldh3a1*, *Galnt3*, *Fgf1*, *Celsr1*, and *Adamts8* increased significantly (*P* < 0.05). Compared with those in the model group, the expression levels of *Runx1*, *Sod2*, and *Tnfaip6* in the sIL-13R*α*2-Fc group increased significantly (*P* < 0.05), whereas the expression levels of *Aldh3a1*, *Galnt3*, *Fgf1*, *Celsr1*, and *Adamts8* in the sIL-13R*α*2-Fc group decreased significantly (*P* < 0.05). The relative expression results were consistent with the sequencing results.

### 3.6. Effect of sIL-13R*α*2-fc on GAG and HA Levels in the Degenerative Intervertebral Disc Tissue of Rats

**Figure 6:**
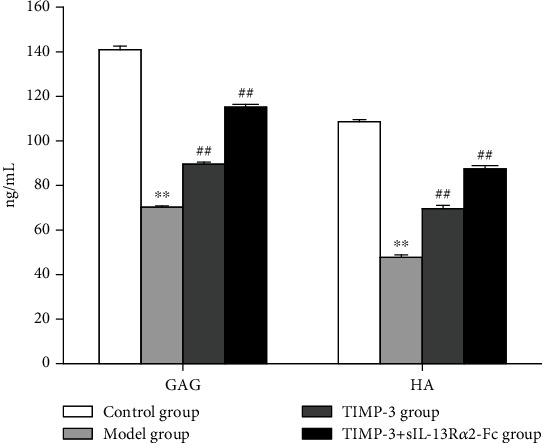
Effect of sIL-13R*α*2-Fc on GAG and HA in the degenerative intervertebral disc tissue of rats determined by ELISA. vs. control group, ^∗∗^*P* < 0.01; vs. model group, ^#^*P* < 0.05, ^##^*P* < 0.01.

We quantitatively measured the GAG and HA levels in degenerative intervertebral disc tissue of rats using an ELISA method, and the results are shown in Figure 6. After 8 weeks of drug intervention, the levels of GAG and HA in the model group were significantly lower than those in the blank control group (*P* < 0.05). The levels of GAG and HA in the TIMP-1 group or the TIMP-1+2 sIL-13R*α*2-Fc group were significantly higher than those in the model group (*P* < 0.05), and those in the TIMP-1+sIL-13R*α*2-Fc group were significantly higher than those in the TIMP-1 group (*P* < 0.05).

### 3.7. Effect of sIL-13R*α*2-fc on the Expression of Type I Collagen and Type II Collagen in the Degenerative Intervertebral Disc Tissue of Rats

**Figure 7:**
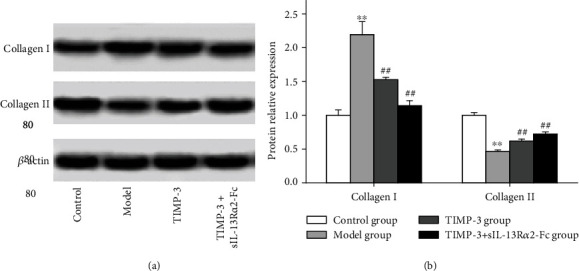
Western blot analysis of the expression of type I collagen and type II collagen in degenerative intervertebral disc tissue. vs. control group, ^∗∗^*P* < 0.01; vs. model group, ^##^*P* < 0.01.

To assess the change in collagen type that occurred during the process of intervertebral disc degeneration, we employed western blotting to measure the expression levels of type I collagen and type II collagen, and the results are shown in Figure 7. After 8 weeks of drug intervention, compared with those in the blank control group, the type I collagen level was significantly higher (*P* < 0.05), whereas the type II collagen level was significantly lower (*P* < 0.05) in the model group. Compared with those in the model group, the type I collagen level in the TIMP-1 group or the TIMP-1+2 sIL-13R*α*2-Fc group was significantly lower (*P* < 0.05), whereas the type II collagen level was significantly higher (*P* < 0.05).

## 4. Discussion

Intervertebral disc degeneration can cause intervertebral disc herniation, neuropathy, and spondylolisthesis, resulting in neck, waist, and leg pain and other symptoms [1]. One of the characteristics of intervertebral disc degeneration is the injury of and formation of lesions in the peripheral tissue, which are difficult to heal. When pathological changes occur in the annulus fibrosus or cartilage endplate, it also means that local tissues have been damaged or have undergone an inflammatory response [2, 4]. The structural change in the intervertebral disc tissue is a sign of impaired intervertebral disc function, and such a structural change is permanent [20]. These structural changes can easily be assessed by both physical and biological means [21, 22]. In this study, we used Sirius red staining to observe the pathological changes in the intervertebral disc tissue of rats. In the model group, the annulus fibrosus of the intervertebral disc tissue was disordered, there was an increased number of breaks, and there was obvious degeneration; however, treatment with sIL-13R*α*2-Fc clearly improved this condition, as evidenced by an evenly arranged annulus fibrosus, a decreased number of breaks, and a lower degree of degeneration. This suggests that sIL-13R*α*2-Fc can reduce the pathological changes in degenerative intervertebral disc tissue.

In this study, we used transcriptome sequencing technology to measure gene expression in the intervertebral disc tissues of the model group and sIL-13R*α*2-Fc treatment group, and we identified 58 differentially expressed genes, of which 22 genes were upregulated and 36 genes were downregulated. In addition, the expression levels of 8 differentially expressed genes in the rat intervertebral disc tissues were verified by RT-PCR. In this study, the RNA-seq method was used to study the effect of sIL-13R*α*2-Fc on the intervertebral disc tissue of rats, and it was found that the most significant differences in the gene expression were among the biological process-related genes, although there were also varying degrees of influence on genes related to cell composition and molecular function. Pathway enrichment analysis revealed that a variety of pathway-related genes were affected. Amino acid, vitamin, and exogenous substance metabolism pathway-related genes showed a downward trend, and polysaccharide biosynthesis pathway genes showed an upward trend. The degeneration of the intervertebral disc begins when the balance between cell generation and apoptosis is disrupted. sIL-13R*α*2-Fc can downregulate metabolism-related genes and upregulate polysaccharide synthesis, which may be one of its important mechanisms of action.

ADAMTS, a depolymerized protein-like metalloproteinase containing a type I platelet binding protein sequence (TSP), is a type of Zn^2+^-dependent secreted metalloproteinase that was discovered after the matrix metalloproteinases (MMPs). ADAMTS is widely expressed in both mammals and invertebrates and can bind to the cell surface and the extracellular matrix through specific protein motifs, thus playing an influential role in pathophysiological processes such as degenerative aging, inflammatory response, tumor growth, and metastasis. ADAMTS is closely involved in a variety of human diseases [23]. Studies have reported the mRNA and protein expression of ADAMTS1, ADAMTS4, ADAMTS5, ADAMTS9, and ADAMTS15 in human intervertebral disc tissue, suggesting that these ADAMTS play a specific role in the normal physiological processes in the intervertebral disc [24]. In a static pressure-induced rat model with caudal intervertebral disc degeneration, the mRNA levels of ADAMTS4, ADAMTS5, ADAMTS7, and ADAMTS12 were found to be increased significantly [25]. ADAMTS degrades proteoglycans during the degeneration of the intervertebral disc, ultimately resulting in the final loss of function of the intervertebral disc [26]. In this study, *Adamts*1, *Adamts*3, *Adamts*4, *Adamts*5, *Adamts*7, *Adamts*9, and *Adamts*15 were not found to be significantly differentially expressed genes in the rat intervertebral disc tissue, *Adamts*8 was found to be significantly downregulated. In addition, the comprehensive GO annotation and KEGG pathway analyses of differentially expressed genes revealed that *Adamts8* is involved in the extracellular matrix signaling pathway, and thus, we further speculate that *Adamts8* may play a role in the process by which sIL-13R*α*2-Fc attenuates intervertebral disc degeneration. The secondary structure of *Adamts8* contains a metal peptidase structure and a zinc ion binding site, showing that it has matrix metalloproteinase activity and the ability to bind metal zinc ions [27]. Studies have shown that the *Adamts8* could have the ability of exhibiting aggrecanase activity and antiangiogenic properties [28, 29]. In this study, the specific mechanism of Adamts8 remains to be further studied.

The imbalance between the synthesis and degradation of the extracellular matrix, resulting in changes in the EMC composition and content, is one of the important characteristics of the intervertebral disc after intervertebral disc degeneration [30, 31]. The ECM in the intervertebral disc is mainly composed of collagen and proteoglycans [32]. The type of collagen in the degenerative intervertebral disc is significantly different from that in a nondegenerative disc, which may reflect the tissue abnormalities that occur during intervertebral disc degeneration and the related repair processes [33]. The changes in the proteoglycan content and composition are considered to be one of the earliest biochemical changes in intervertebral disc degeneration, and they can directly lead to changes in the biomechanical properties of the intervertebral disc [34]. The ADAMTS family is a class of enzymes that play crucial roles in the process of extracellular matrix degradation, and they can degrade almost all the components of the extracellular matrix; accordingly, these enzymes are the most important factors that regulate the dynamic equilibrium of the ECM [35]. Studies have shown that ADAMTS1, ADAMTS4, ADAMTS5, ADAMTS8, ADAMTS9, ADAMTS15, and ADAMTS18 can degrade polyproteoglycans (important components of the nucleus pulposus ECM), and ADAMTS4 and ADAMTS5 are classified as the major polyproteoglycases [36]. Zhang et al. [37] found that the expression levels of ADAMTS7 and ADAMTS12 were upregulated and that the levels of type II collagen were significantly decreased in the lumbar endplate tissue of patients with intervertebral disc degeneration. In our previous study, we found that the ECM content in the degenerative intervertebral disc tissue of rats decreased to varying degrees, the synthesis of type I collagen increased, and the expression of type II collagen decreased, all of these effects were reversed by intervention with sIL-13R*α*2-Fc [18]. In this study, we used the ADAMTS inhibitor TIMP-1 to treat the model rats with intervertebral disc degeneration, and we found that the levels of the ECM components GAG and HA increased, type I collagen expression decreased, and type II collagen expression increased. The levels of the ECM components GAG and HA and the collagen levels in the TIMP-1+2 sIL-13R*α*2-Fc intervention group were significantly higher than those in the TIMP-1 intervention group, indicating that TIMP-1 and sIL-13R*α*2-Fc had a synergistic effect. Incorporating the transcriptome data analysis results, we hypothesize that sIL-13R*α*2-Fc can maintain a dynamic balance in the ECM and delay intervertebral disc degeneration by inhibiting *Adamts8*-mediated ECM degradation.

In summary, the results of this study indicate that sIL-13R*α*2-Fc can inhibit further degradation of the ECM by inhibiting the expression of *Adamts8*, restoring ECM synthesis and degradation to a dynamic equilibrium, and eventually partially slowing the process of intervertebral disc degeneration in rats.

## Figures and Tables

**Table 1:** Primer information.

Gene	Prime
Runx1	Forward: 5′-TCCAGTGCGGTGTATGACTAC-3′
	Reverse:5′-CTTGGAAGCTGCCAGAATGCT-3′
Sod2	Forward: 5′-TGG ACA AAC CTG AGC CCT AA-3′
	Reverse: 5′-GAC CCA AAG TCA CGC TTG ATA-3′
Aldh3a1	Forward: 5′-ACTACATCCTCTGTGACCCC-3′
	Reverse: 5′-GCAAGGTGATGTGGACGATGAC-3′
*Galnt3*	Forward: 5′-GTTGCTAGGAGCAACAGTCGCA-3′
	Reverse: 5′-AGTTCACCGTGGTAGTATTGTAGT-3′
Fgf1	Forward: 5′-GTTCACTTTTCCGCTGCGCC-3′
	Reverse: 5′-ACCCCTCCAGATGCTACACA-3′
Tnfaip6	Forward: 5′-GTCGTCTCGCAACCTACAAGCAG-3′
	Reverse: 5′-CTGACCGTACTTGAGCCGAATGTG-3′
Celsr1	Forward: 5′-CTCTTATTCTTGCCACCACT-3′
	Reverse: 5′-GATTTCTACATTGAGCCCAC-3′
Adamts8	Forward: 5′-TCTGTGACCCCAACAAGAGC-3′'
	Reverse: 5′-CAAACAGTCTCACGCATGGC-3′
GAPDH	Forward: 5′-AGGGCTGCCTTCTCTTGTG-3′
	Reverse: 5′-CCTGGCATTGCCGACA-3′

**Table 2:** Statistical analysis of the number of differentially expressed genes.

Gene ID	Gene symbol	*P* value	log2FC	Description	Regulated
ENSRNOG00000000906	Medag	0.00919	1.040161	Predicted: mesenteric estrogen-dependent adipogenesis protein	Up
ENSRNOG00000001704	Runx1	0	1.091159	Predicted: runt-related transcription factor 1 isoform X1	Up
ENSRNOG00000003923	Rab21	0	1.395875	Ras-related protein Rab-21	Up
ENSRNOG00000007596	Rffl	0	1.141026	Predicted: E3 ubiquitin-protein ligase rififylin isoform X5	Up
ENSRNOG00000011063	Dennd1b	0.000568	1.636745	Predicted: DENN domain-containing protein 1B isoform X2	Up
ENSRNOG00000013618	Ankrd10	0	1.449423	Ankyrin repeat domain-containing protein 10	Up
ENSRNOG00000018450	Slc25a22	0	5.092963	Predicted: mitochondrial glutamate carrier 1 isoform X1	Up
ENSRNOG00000019048	Sod2	0	1.234773	Superoxide dismutase (Mn), mitochondrial precursor	Up
ENSRNOG00000021119	Pdcd2l	0.001631	1.483934	Programmed cell death protein 2-like	Up
ENSRNOG00000015002	Abhd15	0.003322	1.851272	Protein ABHD15 precursor	Up
ENSRNOG00000025670	Shisa3	0.0014	1.954814	Protein shisa-3 homolog precursor	Up
ENSRNOG00000027837	Gm14569	0.005741	1.751623	Similar to novel protein (predicted), partial	Up
ENSRNOG00000036662	Wdr45b	0	1.102958	WD repeat domain phosphoinositide-interacting protein 3	Up
ENSRNOG00000045941	Susd6	0.000112	1.030092	Predicted: sushi domain-containing protein 6 isoform X1	Up
ENSRNOG00000049185	LOC100911032	0.006145	1.808194	Immunoglobulin light chain, partial	Up
ENSRNOG00000050792	Tnfaip6	0.001631	1.561718	Tumor necrosis factor-inducible gene 6 protein precursor	Up
ENSRNOG00000052100	Eddm3b	0	2.071116	rCG61219	Up
Rattus_norvegicus_newGene_23468	—	0.008698	1.747348	mCG61979, isoform CRA_b, partial	Up
Rattus_norvegicus_newGene_26511	—	0.00812	1.748733	Predicted: uncharacterized protein LOC108348366 isoform X1	Up
Rattus_norvegicus_newGene_26728	—	0.009034	1.689907	—	Up
Rattus_norvegicus_newGene_37488	—	0.008019	1.609688	Predicted: neuron navigator 3 isoform X2 (Microtus ochrogaster)	Up
Rattus_norvegicus_newGene_47258	—	0	2.665634	rCG63686	Up
ENSRNOG00000010107	Palld	0	-1.176578	Predicted: palladin isoform X2	Down
ENSRNOG00000011096	LOC100912369	0.000218	-2.164710	High-mobility group protein B3-like isoform X1	Down
ENSRNOG00000011151	Tenm4	0.005755	-1.357832	Teneurin-4	Down
ENSRNOG00000011595	Senp8	0.001631	-1.611544	Sentrin-specific protease 8	Down
ENSRNOG00000013867	Fgf1	0.008019	-1.262288	Predicted: fibroblast growth factor 1 isoform X3	Down
ENSRNOG00000014590	Stk33	0.004748	-1.806869	Predicted: serine/threonine-protein kinase 33 isoform X1	Down
ENSRNOG00000015550	Ptgds	0	-2.222049	Prostaglandin-7H2 D-isomerase precursor	Down
ENSRNOG00000015636	Wdr34	0	-1.482727	WD repeat-containing protein 34	Down
ENSRNOG00000016769	Rab38	0.007215	-1.475357	Ras-related protein Rab-38	Down
ENSRNOG00000017233	Mmachc	0.006781	-1.017858	Methylmalonic aciduria and homocystinuria type C protein	Down
ENSRNOG00000002331	Aldh3a1	0.008355	-1.687169	Aldehyde dehydrogenase, dimeric NADP-preferring	Down
ENSRNOG00000002579	Parm1	0	-1.803845	Castration-induced prostatic apoptosis-related protein 1	Down
ENSRNOG00000003031	Atp2b4	0.000242	-1.261295	Predicted: plasma membrane calcium-transporting ATPase 4 isoform X1	Down
ENSRNOG00000005535	Ikzf4	0.004819	-1.501689	Predicted: zinc finger protein Eos isoform X1	Down
ENSRNOG00000005574	Adamts8	0.004807	-1.664895	Predicted: A disintegrin and metalloproteinase with thrombospondin motifs 8	Down
ENSRNOG00000005727	Galnt3	0	-1.437957	Polypeptide N-acetylgalactosaminyltransferase 3	Down
ENSRNOG00000009965	Pih1d2	0	-2.237542	PIH1 domain-containing protein 2	Down
ENSRNOG00000021285	Celsr1	0.008019	-1.757083	Predicted: cadherin EGF LAG seven-pass G-type receptor 1 isoform X4	Down
ENSRNOG00000024237	Scel	0.000154	-2.031804	Predicted: sciellin isoform X1	Down
ENSRNOG00000024793	Kctd21	0.000107	-1.933313	Predicted: BTB/POZ domain-containing protein KCTD21	Down
ENSRNOG00000026211	Mri1	0.001893	-1.404079	Methylthioribose-1-phosphate isomerase	Down
ENSRNOG00000027590	Jakmip3	0.000471	-2.080541	Janus kinase and microtubule-interacting protein 3	Down
ENSRNOG00000029022	Zfp112	0.002058	-1.570479	Predicted: zinc finger protein 112 isoform X1	Down
ENSRNOG00000036673	Sectm1b	0.009235	-1.586613	Secreted and transmembrane protein 1 precursor	Down
ENSRNOG00000039969	Dsc2	0.006781	-1.657599	Desmocollin-2 precursor	Down
ENSRNOG00000043267	Mrm2	0.000159	-1.505554	rRNA methyltransferase 2, mitochondrial	Down
ENSRNOG00000051238	Mphosph8	0.006353	-1.709101	M-phase phosphoprotein 8	Down
ENSRNOG00000057827	—	0.001631	-1.744961	Predicted: uncharacterized protein RGD1560492 isoform X2	Down
Rattus_norvegicus_newGene_5312	—	0.0014	-1.983142	mCG1045525, partial	Down
Rattus_norvegicus_newGene_7480	—	0	-3.134018	mCG145399, partial	Down
SRattus_norvegicus_newGene_7720	—	0.001595	-1.398793	rCG35828	Down
Rattus_norvegicus_newGene_14927	—	0	-2.505698	Ac1071	Down
Rattus_norvegicus_newGene_28112	—	0.000159	-1.719767	—	Down
Rattus_norvegicus_newGene_39936	—	0.00156	-1.964885	Predicted: protein turtle homolog B isoform X1	Down
Rattus_norvegicus_newGene_40776	—	0.001055	-2.016483	—	Down
Rattus_norvegicus_newGene_40778	—	0.000216	-2.174655	mCG1028420, partial	Down

**Table 3:** Statistical table of differentially expressed genes in significantly enriched KEGG pathways.

Gene name	Gene symbol	Entry	Log2FC	Regulated
ENSRNOG00000001704	Runx1	K08367	1.091159	Up
ENSRNOG00000019048	Sod2	K04564	1.234773	Up
ENSRNOG00000002331	Aldh3a1	K00129	-1.687169	Down
ENSRNOG00000003031	Atp2b4	K05850	-1.261295	Down
ENSRNOG00000005727	Galnt3	K00710	-1.437957	Down
ENSRNOG00000013867	Fgf1	K18496	-1.262288	Down
ENSRNOG00000026211	Mri1	K08963	-1.404079	Down
ENSRNOG00000039969	Dsc2	K07601	-1.657599	Down
ENSRNOG00000017233	Mmachc	K14618	-1.017858	Down
ENSRNOG00000005574	Adamts8	K08623	-1.664895	Down
ENSRNOG00000015550	Ptgds	K01830	-2.222049	Down

## Data Availability

The datasets supporting the conclusions of this article are included within the article.

## Abbreviations

sIL-13R*α*2-Fc: Soluble interleukin-13 R alpha2 receptor fusion protein
ECM: Extracellular matrix
ADAMTS: A depolymerized protein-like metalloproteinase containing a type I platelet binding protein sequence
IL-13: Interleukin-13
GAG: Glycosaminoglycan
CS: Chondroitin sulfate
KS: Keratan sulfate
HA: Hyaluronic
OD: Optical density
RT-PCR: Reverse transcription-polymerase chain reaction.
